# Spatiotemporal DNA methylation dynamics shape megabase-scale methylome landscapes

**DOI:** 10.26508/lsa.202302403

**Published:** 2024-01-17

**Authors:** Hidehiro Toh, Hiroyuki Sasaki

**Affiliations:** 1 Advanced Genomics Center, National Institute of Genetics , Mishima, Japan; 2 Division of Epigenomics and Development, Medical Institute of Bioregulation, Kyushu University , Fukuoka, Japan

## Abstract

DNA methylation is an essential epigenetic mechanism that regulates cellular reprogramming and development. Studies using whole-genome bisulfite sequencing have revealed distinct DNA methylome landscapes in human and mouse cells and tissues. However, the factors responsible for the differences in megabase-scale methylome patterns between cell types remain poorly understood. By analyzing publicly available 258 human and 301 mouse whole-genome bisulfite sequencing datasets, we reveal that genomic regions rich in guanine and cytosine, when located near the nuclear center, are highly susceptible to both global DNA demethylation and methylation events during embryonic and germline reprogramming. Furthermore, we found that regions that generate partially methylated domains during global DNA methylation are more likely to resist global DNA demethylation, contain high levels of adenine and thymine, and are adjacent to the nuclear lamina. The spatial properties of genomic regions, influenced by their guanine–cytosine content, are likely to affect the accessibility of molecules involved in DNA (de)methylation. These properties shape megabase-scale DNA methylation patterns and change as cells differentiate, leading to the emergence of different megabase-scale methylome patterns across cell types.

## Abstract (precis)

The guanine–cytosine content of genomic regions influences their spatial properties, resulting in various megabase-scale methylome patterns across different cell types.

## Introduction

DNA methylation is a critical epigenetic modification that regulates cellular processes. Whole-genome bisulfite sequencing (WGBS) is the primary technique for studying global DNA methylation patterns, or methylomes, at single-base resolution (Olova et al, 2018). This method has revealed partially methylated domains (PMDs), which span hundreds of kilobases (kb) and exhibit moderate-to-low methylation levels (Lister et al, 2009). PMDs often spatially coincide with lamina-associated domains (LADs), DNA regions that interact with the nuclear lamina and contain adenine/thymine (A/T)-rich sequences (Guelen et al, 2008; Berman et al, 2011; Meuleman et al, 2013). In addition, the boundaries of PMDs are close to those of topologically associated domains (Salhab et al, 2018). Furthermore, within the nucleus, chromosomes occupy unique regions called chromosome territories (Cremer et al, 2001). For instance, in humans, the A/T-rich chromosome (Chr) 18 resides near the nuclear lamina, whereas the guanine/cytosine (G/C)-rich Chr19 is located near the nuclear center (Kind et al, 2015; Tan et al, 2018; Girelli et al, 2020). These findings suggest that within a region of the mammalian genome, there is a relationship between DNA methylation levels, G/C content, 3D chromosome structure, and spatial distribution within the nucleus.

Differences in methylation levels between A/T-rich and G/C-rich regions have been observed to vary by cell type. Somatic cells and embryonic stem cells show higher methylation in A/T-rich regions, in contrast to placenta, IMR-90 fibroblasts, and cancer cell lines, which show higher methylation in G/C-rich regions (Lister et al, 2009; Hansen et al, 2011; Hon et al, 2012, 2013; Schroeder et al, 2013; Schultz et al, 2015). It has been suggested that genomic base composition may influence the formation of distinct methylome profiles during cellular differentiation (Quante & Bird, 2016; Liu et al, 2018). However, this interplay between DNA methylation and genomic base composition has not been fully elucidated.

In this study, we analyzed 559 publicly available WGBS datasets, tracking the methylome dynamics during different stages of tissue development and cell differentiation in humans and mice. Our comprehensive investigation reveals a spatiotemporal dynamic process that largely contributes to the emergence of distinct megabase-scale methylome patterns across cell types.

## Results

### Megabase-scale methylome patterns and their association with global methylation events

**Figure S1.**
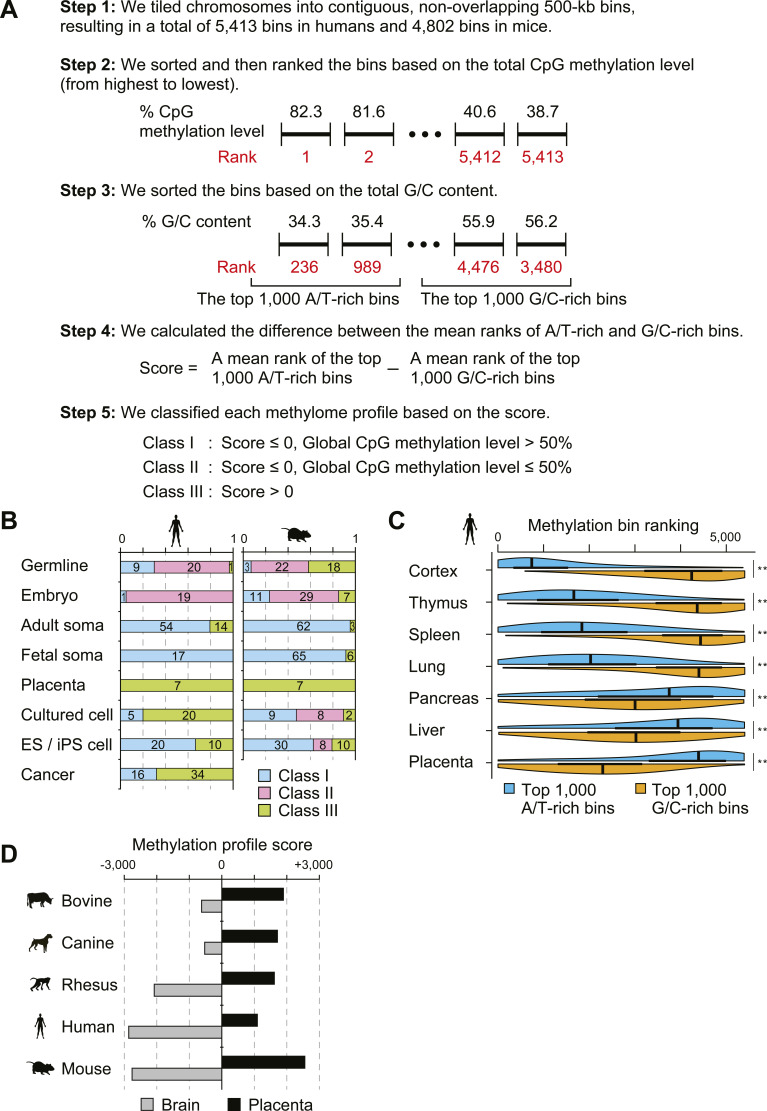
Classification strategy and distribution of methylome profiles across cell types and species. **(A)** Strategy used to categorize methylome profiles in this study. **(B)** Proportion of the three classes for each cell type. **(C)** Variation in DNA methylation levels between G/C-rich and A/T-rich bins in adult human somatic tissues. Tissues are ordered by increasing the methylation score, and bin ranks for the top 1,000 G/C-rich (orange) and A/T-rich (blue) regions are shown in half-violin plots. High-ranking bins correspond to higher levels of DNA methylation in each methylome. Statistical significance was assessed using the Wilcoxon rank sum test, with double asterisks (**) indicating *P* < 2 × 10^−16^. The whole-genome bisulfite sequencing data used in this plot were acquired from accession numbers GSE46644 (cortex), CRA000114 (placenta), and GSE16256 (all other tissues). **(D)** Methylome profiling in the brain and placenta of five mammalian species. The methylome profiles of bovine, canine, and rhesus brain and placenta were classified using methods analogous to those used in humans and mice. The whole-genome bisulfite sequencing data used in this plot were acquired from accession numbers GSE63330, GSE77124, and GSE106538.

**Figure 1.**
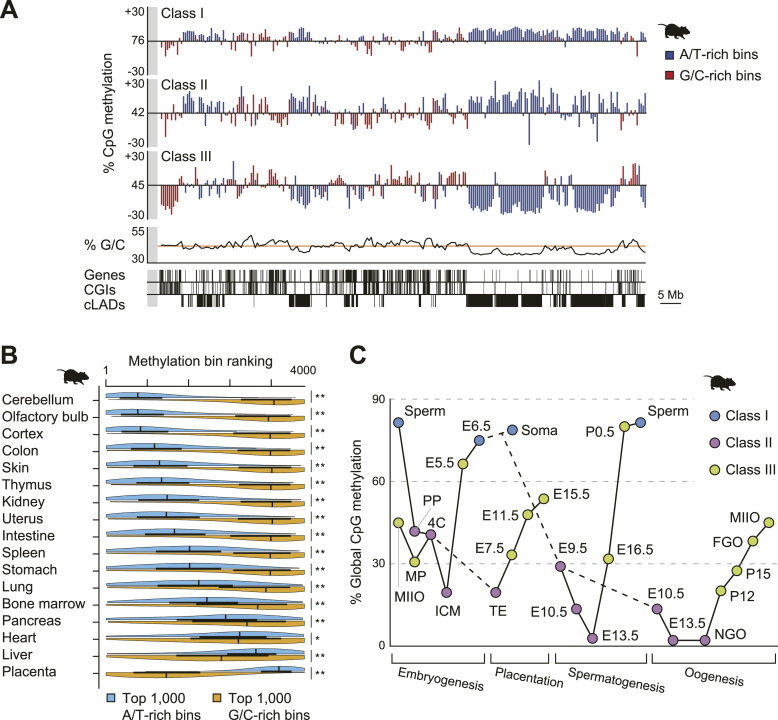
Classification of megabase-scale methylome patterns in mouse cells and tissues. **(A)** Representative methylome patterns for each of the three classes. The overall CpG methylation levels in each 500-kb bin on mouse chromosome 14 are displayed for Class I (cerebellum), Class II (paternal pronucleus), and Class III (E5.5 visceral endoderm) profiles, with the y-axis adjusted to the global CpG methylation level for each tissue. Bins with high G/C content (>41.9%) and low G/C content (≤41.9%) are denoted by red and blue lines, respectively. The G/C content of the 500-kb bins is also illustrated, with an orange line representing the mean of the autosomes. The distribution of RefSeq genes, CpG islands, and constitutive lamina-associated domains is shown at the bottom. **(B)** Differential DNA methylation levels between G/C-rich and A/T-rich bins in somatic tissues of adult mice. The tissues are arranged from the lowest to the highest score, and the ranks of the top 1,000 G/C-rich (orange) and top 1,000 A/T-rich (blue) bins are displayed in half-violin plots. High-ranking bins correspond to higher levels of DNA methylation in each methylome. Statistical analysis was conducted using the Wilcoxon rank sum test; symbols denote statistical significance, with * indicating *P* < 0.05 and ** indicating *P* < 2 × 10^−16^. Data for this plot are derived from Hon et al (2013). **(C)** Dynamics of mouse methylome reprogramming during embryogenesis and gametogenesis. The y-axis denotes the global CpG methylation level for each cell and tissue. Blue, pink, and green circles indicate Class I, II, and III methylome profiles, respectively. The values beginning with E and P represent the number of embryonic and postnatal days, respectively. MP, maternal pronucleus; PP, paternal pronucleus; 4C, four-cell embryo; ICM, inner cell mass; TE, trophectoderm; NGO, non-growing oocyte; FGO, fully grown oocyte; MIIO, metaphase II oocyte.

We analyzed base-resolution methylome maps for 258 human and 301 mouse samples from publicly available data to classify methylome patterns at the megabase scale (Tables S1 and S2). We divided chromosomes into non-overlapping 500-kb bins to capture megabase-scale methylation changes. We then classified the methylome profiles into three classes based on the difference in methylation levels between the top 1,000 G/C-rich and top 1,000 A/T-rich bins (Fig S1A). Class I and II profiles showed higher methylation levels in A/T-rich bins than in G/C-rich ones, with global C-G dinucleotide (CpG) methylation levels above and below 50%, respectively. In contrast, Class III profiles displayed an “inverted” pattern, with higher methylation levels in G/C-rich bins than in A/T-rich ones (Fig 1A).

Table S1 Human whole-genome bisulfite sequencing data used in this study.

Table S2 Mouse whole-genome bisulfite sequencing data used in this study.

Class I profiles were predominantly found in somatic tissues (Fig S1B); ectoderm-derived tissues exhibited greater methylation differences between G/C-rich and A/T-rich bins (Figs 1B and S1C), as well as a higher proportion of highly methylated domains (HMDs) in A/T-rich regions. Class II profiles were detected in preimplantation embryos and early primordial germ cells (PGCs), both undergoing global DNA demethylation (Fig 1C). Class III profiles were found in trophoblasts, early epiblasts, prospermatogonia, and oocytes, all undergoing global DNA methylation (Fig 1C). This class also included placenta, fibroblasts, and cultured cancer cell lines (Fig S1B), which are known to contain PMDs (Lister et al, 2009; Hansen et al, 2011; Hon et al, 2012; Schroeder et al, 2013). These results illustrate a clear relationship between megabase-scale methylome patterns and global DNA (de)methylation events. In other mammals, including bovine, canine, and rhesus, the brain and placenta methylome profiles were similarly classified as Class I and III, respectively (Fig S1D). This suggests a potentially universal mechanism for establishing megabase-scale methylome patterns across mammalian tissues.

### Dynamics of DNA methylation during development in humans and mice: insights from G/C-rich and A/T-rich regions

**Figure 2.**
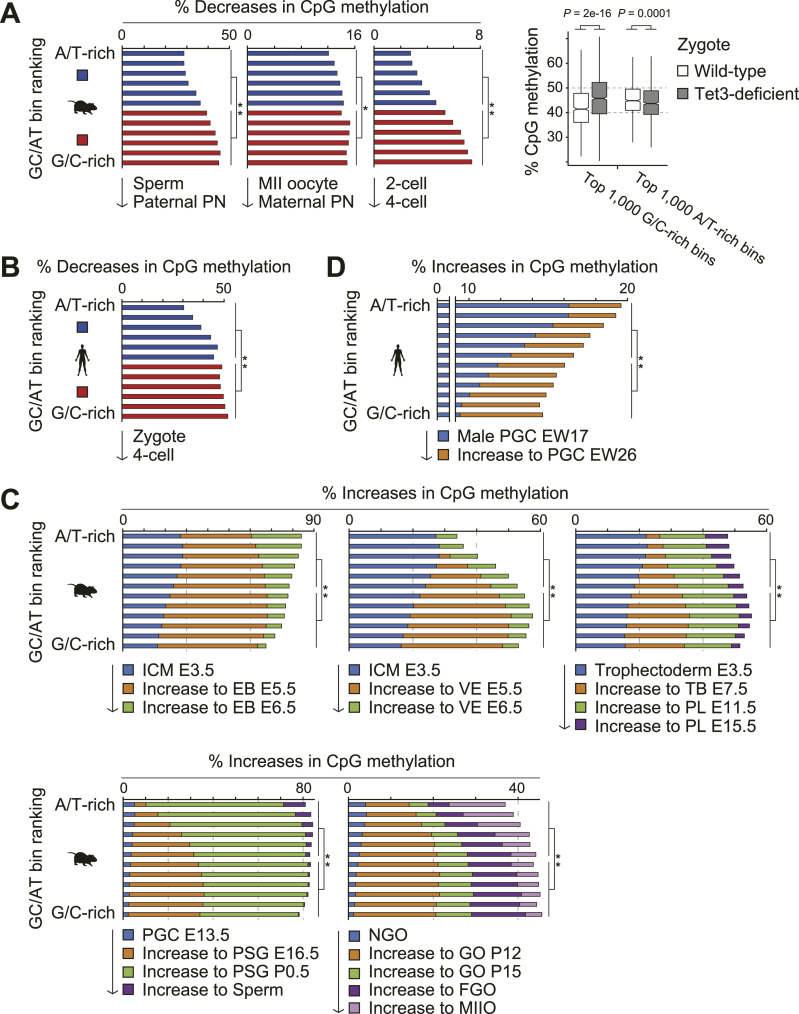
Dynamics of DNA methylation in G/C-rich and A/T-rich regions during reprogramming. All non-overlapping 500-kb bins were categorized into 12 groups based on their G/C content. Each group consists of 450 bins for humans and 400 bins for mice. **(A, B)** Decreases in DNA methylation levels between indicated developmental stages in mice (A) and humans (B). The overall CpG methylation levels in each G/C-rich and A/T-rich bin group are shown as red and blue bars, respectively. The statistical significance of these differences was assessed using the exact Wilcoxon rank sum test; asterisks indicate *P* < 0.01 (*) and *P* < 0.005 (**). **(A)** Box plots illustrate CpG methylation levels of WT (white) and TET3-deficient (gray) zygotes in the top 1,000 G/C-rich and A/T-rich bins ((A), right), with the data for this plot derived from Peat et al (2014). In these box plots, the median is shown by the central line, the 25th and 75th percentiles are shown by the box, and the whiskers extend up to 1.5 times the interquartile range. *P*-values were calculated using the Wilcoxon rank sum test. PN, pronucleus; MII oocyte, metaphase II oocyte. **(C, D)** Increases in DNA methylation levels between indicated developmental stages in mice (C) and humans (D). Blue bars represent baseline CpG methylation levels in the reference cell type. Colored bars above the blue indicate additional CpG methylation compared with this baseline. Increases in methylation levels in the second stage (indicated by orange bars) were evaluated for statistical significance using the exact Wilcoxon rank sum test; double asterisks (**) indicate *P* < 0.005. The values beginning with E and P represent the number of embryonic and postnatal days, respectively. ICM, inner cell mass; EB, epiblast; VE, visceral endoderm; TB, trophoblast; PL, placenta; PSG, prospermatogonia; NGO, non-growing oocyte; GO, growing oocyte; FGO, fully grown oocyte; MIIO, metaphase II oocyte; EW, embryonic weeks.

**Figure S2.**
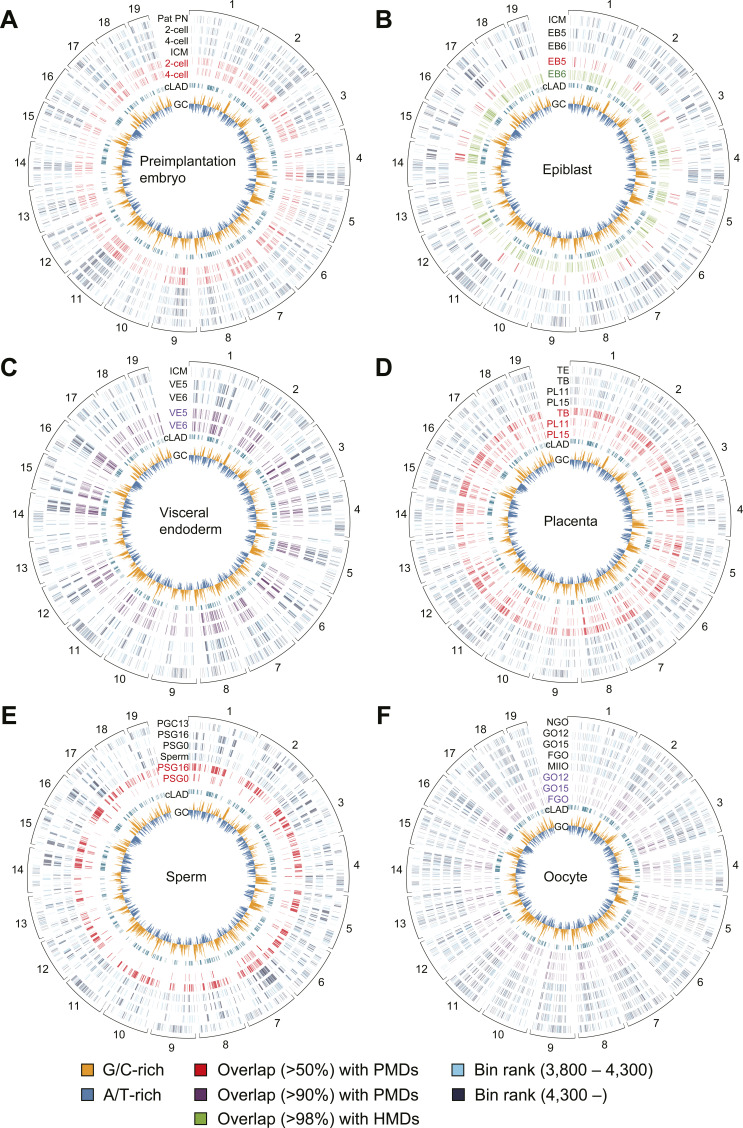
Circular representation of methylome dynamics during mouse reprogramming. **(A, B, C, D, E, F)** This figure displays DNA methylation levels in 500-kb bins across autosomes for various mouse cell lineages during reprogramming, including the preimplantation embryo (A), epiblast (B), visceral endoderm (C), placenta (D), sperm (E), and oocyte (F) lineages. The innermost circle indicates the G/C content (GC) of the bins, with higher values indicated by the outermost regions. The next circle represents bins with more than 70% overlap with constitutive lamina-associated domains. The red and purple circles illustrate bins with more than 50% and 90% overlap with partially methylated domains, respectively. **(B)** Green circles in panel (B) represent bins with more than 98% overlap with highly methylated domains, whereas blue circles correspond to bins ranked 3,800 or less (i.e., hypomethylated regions). The outermost values are the chromosome numbers. Pat PN, paternal pronucleus; ICM, inner cell mass; EB5, E5.5 epiblast; EB6, E6.5 epiblast; VE5, E5.5 visceral endoderm; VE6, E6.5 visceral endoderm; TE, trophectoderm; TB, E7.5 trophoblast; PL11, E11.5 placenta; PL15, E15.5 placenta; PGC13, E13.5 primordial germ cell; PSG16, E16.5 prospermatogonia; PSG0, P0.5 prospermatogonia; NGO, non-growing oocyte; GO12, P12 growing oocyte; GO15, P15 growing oocyte; FGO, fully grown oocyte; MIIO, metaphase II oocyte.

During mammalian development, the epigenome undergoes reprogramming through two distinct waves, as illustrated in Fig 1C (Shirane & Lorincz, 2022). We examined the methylome dynamics during global DNA demethylation. In mice, during the first reprogramming, G/C-rich regions were more demethylated than A/T-rich regions, particularly in the paternal pronucleus (Figs 2A and S2A). This demethylation, mediated by the DNA dioxygenase TET3, occurred predominantly in G/C-rich regions at the zygotic stage (Fig 2A, right). These regions were more demethylated during the transition from two-cell to four-cell embryos (Fig 2A). During the second reprogramming, A/T-rich regions showed greater resistance to global DNA demethylation (Fig 1C). In humans, G/C-rich regions were more demethylated during the transition from zygotes to four-cell embryos (Fig 2B). In addition, A/T-rich regions had higher methylation levels in early preimplantation embryos and early PGCs (Table S1).

We also examined the methylome dynamics during global DNA methylation. In mice, G/C-rich regions were more methylated in early epiblasts (up to embryonic day 5.5), visceral endoderm, trophoblasts, prospermatogonia, and growing oocytes (Figs 2C and S2B–F). In humans, G/C-rich regions were more methylated in late male PGCs from 17 to 26 embryonic weeks (Fig 2D). These findings not only demonstrate that G/C-rich regions are highly susceptible to both global DNA demethylation and methylation during reprogramming in mice and humans, but also suggest that this susceptibility strongly influences the formation of megabase-scale methylome patterns.

### Formation and characteristics of PMDs in different mouse tissues

**Figure 3.**
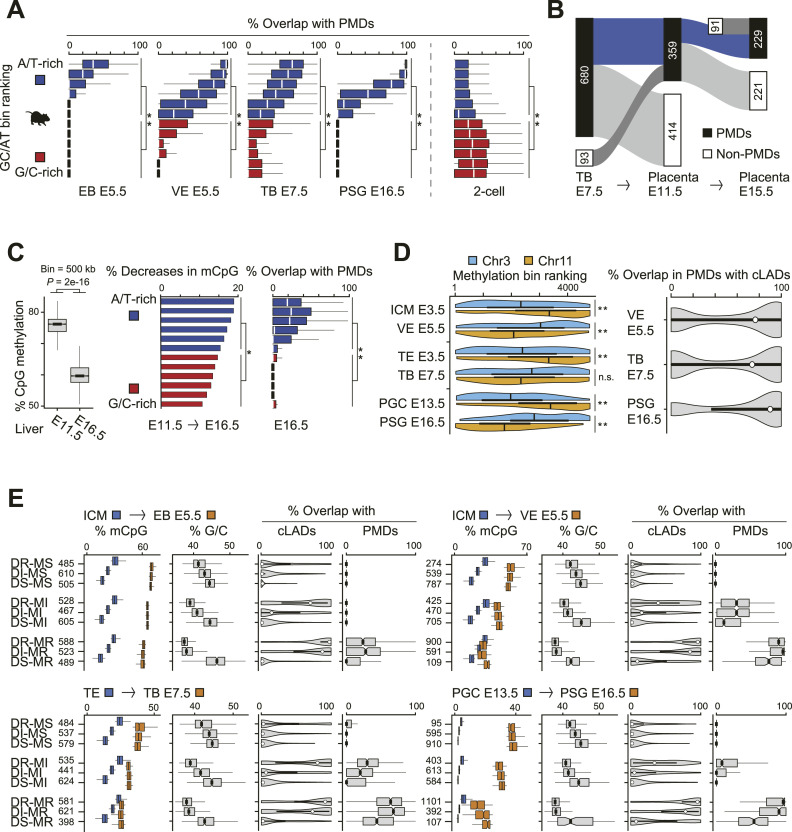
Partially methylated domain (PMD) dynamics and their association with changes in global methylation during mouse development. **(A)** Distribution of PMDs in genomic regions with varying G/C content. Box plots show the proportion of PMDs overlapping with bins within each of the 12 groups defined in Fig 2A. Statistical significance was assessed using the exact Wilcoxon rank sum test; double asterisks (**) indicate *P* < 0.005. EB, epiblast; VE, visceral endoderm; TB, trophoblast; PSG, prospermatogonia. **(B)** PMD reduction during placental development. The numbers in the boxes indicate the total size (in megabases) of PMDs at each stage. **(C)** DNA demethylation in fetal liver. Box plots depict global CpG methylation levels of fetal liver (left, N = 4,802), and *P*-values were calculated with the Wilcoxon rank sum test. Bars indicate decreases in DNA methylation levels in each G/C-rich and A/T-rich bin group from E11.5 to E16.5 (middle), whereas box plots display the proportion of overlap between PMDs and bins in each group (right), where the 12 bin groups are the same as in panel (A). Statistical significance was determined by the Wilcoxon rank sum test; asterisks indicate *P* < 0.005 (*) and *P* < 0.0001 (**). **(D)** Influence of chromosome territories on DNA methylation levels in mice. The half-violin plots illustrate the ranks of bins in Chr11 (orange, N = 238) and Chr3 (blue, N = 314), where higher ranked bins correspond to higher levels of DNA methylation (left). The violin plots display the overlap rate between PMDs and constitutive lamina-associated domains in VE (N = 2,976), TB (N = 3,739), and PSG (N = 2,055) (right). Statistical analysis was performed using the Wilcoxon rank sum test; double asterisks (**) indicate *P* < 0.0001. ICM, inner cell mass; TE, trophectoderm. **(E)** Characteristics of the nine genomic bin groups. The nine bin groups are a combination of the three groups (each comprising 1,600 bins) classified based on methylation levels in two states of each lineage (see the Materials and Methods section for details). The number of bins, CpG methylation levels (mCpG), G/C content, and overlap rate with constitutive lamina-associated domains and PMDs in each bin group are shown. DR, demethylation-resistant; DI, demethylation-intermediate; DS, demethylation-susceptible; MS, methylation-susceptible; MI, methylation-intermediate; MR, methylation-resistant. **(A, C, E)** Note: For all box plots in (A, C, E), the center line indicates the median, the edges of the box represent the 25th and 75th percentiles, and the whiskers extend to 1.5 times the interquartile range.

**Figure S3.**
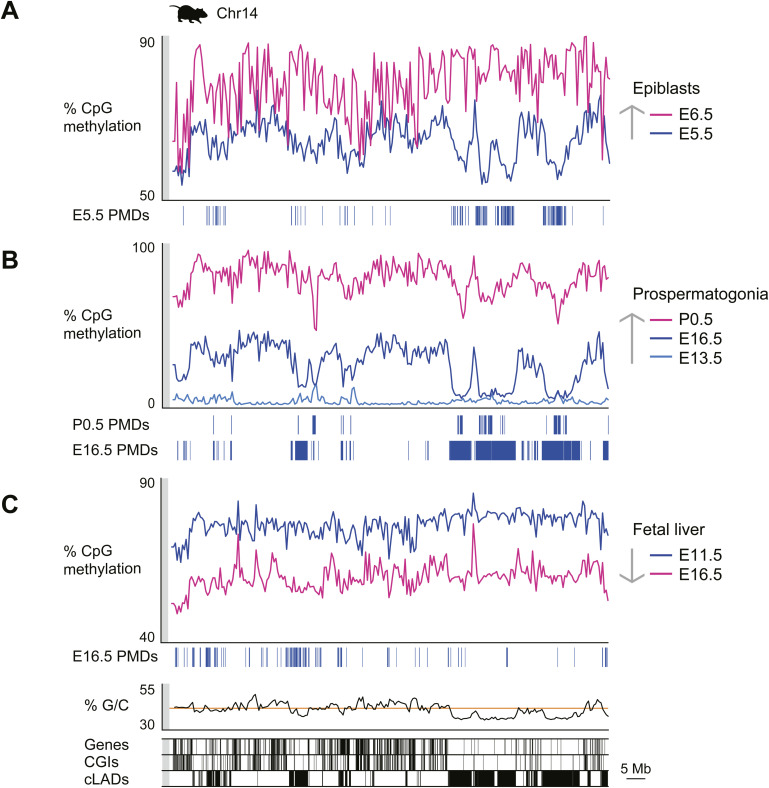
Methylome patterns during mouse cell development and tissue differentiation. **(A, B, C)** Overall CpG methylation levels in each 500-kb bin on mouse chromosome 14 are displayed for epiblasts (A), prospermatogonia (B), and fetal liver (C) profiles. Partially methylated domains are shown as blue rectangles. The G/C content of the 500-kb bins is also illustrated, with an orange line representing the mean of the autosomes. The distribution of RefSeq genes, CpG islands, and constitutive lamina-associated domains is shown at the bottom. The values beginning with E and P represent the number of embryonic and postnatal days, respectively.

**Figure 4.**
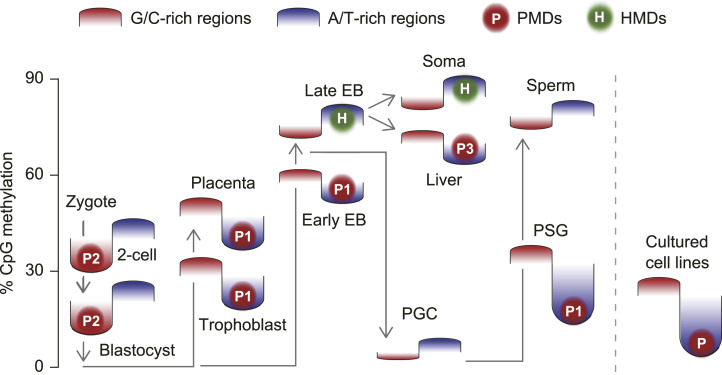
Schematic overview of DNA methylation changes in G/C-rich and A/T-rich regions across development. Red and blue regions indicate DNA methylation levels within G/C-rich and A/T-rich regions in cells and tissues, respectively, whereas arrows indicate cell differentiation pathways. In this figure, the partially methylated domains generated by the first, second, and third patterns described in the Discussion section are shown as P1, P2, and P3, respectively. EB, epiblast; PSG, prospermatogonia.

We examined the formation of PMDs in various mouse tissues. PMDs were predominantly observed in A/T-rich regions during global DNA methylation (Figs 3A and S2B–E). In the placenta, trophoblast-derived PMDs gradually acquired methylation during development (Fig 2C), resulting in a reduction in total PMD area (Fig 3B). Similarly, the methylomes of epiblasts and prospermatogonia also showed a reduction in total PMD area as cells differentiated (Fig S3A and B). In contrast, in cases such as fetal liver, the formation of PMDs was more pronounced in A/T-rich regions during global DNA demethylation, which differed from the pattern observed in other tissues described above (Figs 3C and S3C). In addition, the formation of PMDs in G/C-rich regions was observed during reprogramming, as shown in two-cell and four-cell embryos (Figs 3A and S2A). Thus, we identified three potential patterns that may underlie the in vivo generation of PMDs (Fig 4).

To examine the effect of chromosome territories on DNA methylation levels in mice (Mayer et al, 2005; Stevens et al, 2017), we used constitutive LADs as indicators, which have been shown to be conserved across cell types (Peric-Hupkes et al, 2010). Specifically, we focused on Chr11 and Chr3, which contain the least and most constitutive LADs, respectively. Compared with Chr3, Chr11 was more demethylated in a globally hypomethylated state, but subsequently was more methylated during global DNA methylation (Fig 3D). These findings suggest that regions located near the nuclear center are highly susceptible to global DNA demethylation and methylation during reprogramming.

We classified 500-kb genomic bins into nine groups based on their methylation levels across different developmental lineages (see the Materials and Methods section for details) and then evaluated the distinct characteristics of each group (Fig 3E). During the transition from a state of global hypomethylation to one of the globally increased methylation levels, regions (DR-MR) resistant to both global DNA demethylation and methylation showed higher A/T content and overlapped more with constitutive LADs and PMDs (Fig 3E). These observations showed that regions generating PMDs during global DNA methylation are more likely to resist global DNA demethylation, probably because of their condensed chromatin structure near the nuclear lamina.

## Discussion

Previous studies have extensively investigated specific cell methylome profiles, but less has been done to understand the underlying processes that shape these profiles. Therefore, in this study, we aimed to elucidate these processes by tracking methylome dynamics during tissue development and cellular differentiation.

Hi-C analyses have identified two distinct megabase-scale genomic regions. Compartment A is G/C-rich, euchromatic, and located near the nuclear center, whereas compartment B is A/T-rich, heterochromatic, and located near the nuclear lamina (Lieberman-Aiden et al, 2009; Nagano et al, 2017; Stevens et al, 2017). Compartment A shows lower methylation levels in preimplantation embryos (Ke et al, 2017; Quan et al, 2021), and PMDs associate with compartment B in visceral endoderm (Zhang et al, 2018). Compartment A/B formation precedes DNA methylation, and DNA methyltransferase DNMT3 and DNA dioxygenase TET show increased accessibility in compartment A (Nothjunge et al, 2017). It is hypothesized that regions that replicate early in the S phase, such as compartment A, have increased exposure to methylation maintenance machinery, leading to more extensive methylation than regions that replicate later (Zhou et al, 2018). Our results, together with previous reports, suggest that the machinery responsible for DNA methylation and demethylation is more accessible to compartment A during reprogramming. As a result, G/C-rich regions, which are closer to the nuclear center and occupy a large part of compartment A (Bouwman et al, 2023), may be more susceptible to changes in global methylation. In contrast, the condensed chromatin structure of many A/T-rich regions may increase their resistance to changes in global methylation during reprogramming.

Our study suggests that the spatial characteristics of G/C-rich and A/T-rich regions, as described above, strongly influence the formation of megabase-scale methylome patterns during reprogramming (Fig 4). Furthermore, A/T-rich regions are more methylated from early to late epiblasts and thus have higher methylation levels in late epiblasts. This methylation pattern is subsequently transferred to fetal somatic tissues and remains evident in most adult tissues. Thus, DNA regions near the nuclear center become less susceptible to global methylation and demethylation as differentiation proceeds. Presumably, this change in susceptibility contributes to the establishment of stable gene expression patterns and cellular identity. Conversely, in the placenta, methylation levels in G/C-rich regions remain higher than those in A/T-rich regions, and their global methylation levels are lower than in normal somatic cells; as discussed in the mouse trophoblast stem cell study (Weigert et al, 2023), further studies are needed in this regard.

Although the exact cause of the in vivo generation of PMDs remains elusive (Schroeder et al, 2013), our results suggest that the spatial characteristics of G/C-rich and A/T-rich regions influence the in vivo generation of PMDs and that there may be three distinct generation patterns (Fig 4). The first pattern is observed during global DNA methylation, particularly in A/T-rich regions near the nuclear lamina, where methylation progresses more slowly. Over time, these PMDs may decrease or disappear, depending on their size and the histone H3 marks within the domains. The second pattern is observed during global DNA demethylation, particularly in G/C-rich regions, where demethylation is more accelerated. The third pattern occurs during differentiation into specific somatic cells such as fetal liver, T cells, and erythroblasts (Durek et al, 2016; Bartholdy et al, 2018; He et al, 2020). This pattern, characterized by increased demethylation in A/T-rich regions, may account for PMDs observed in primary tumor methylomes (Brinkman et al, 2019). In cultured cell lines, PMDs have also been detected in A/T-rich regions, possibly because of insufficient methylation maintenance during prolonged cell passage (Cruickshanks et al, 2013; Du et al, 2021; Endicott et al, 2022). Thus, PMDs in A/T-rich regions have been frequently observed both in vivo and in vitro (Gaidatzis et al, 2014; Salhab et al, 2018; Zhou et al, 2018; Decato et al, 2020), but they may be generated in different patterns as described above.

We used WGBS datasets generated using different protocols, sequencers, and sequencing depths, and in different laboratories. Previous studies have shown that several factors can affect the accuracy of estimating DNA methylation levels in WGBS (Toh et al, 2017; Olova et al, 2018; Raine et al, 2018). We observed that although this leads to variation in DNA methylation levels even within the same tissue and cell-type datasets, they consistently show similar methylome patterns at the megabase scale. This consistency supports the reliability of our conclusions drawn from the publicly available WGBS datasets. However, for comprehensive validation, future studies should ideally use standardized WGBS protocols to confirm these findings.

In this study, by analyzing 559 WGBS datasets, we have highlighted the critical role of spatial features and temporal dynamics associated with G/C content in shaping megabase-scale methylome patterns. The mechanisms by which these spatial features and temporal dynamics influence cell reprogramming and development are expected to be elucidated in detail in future studies.

## Materials and Methods

### Acquisition of publicly available WGBS data

All methylome maps used in this study were generated from publicly available raw fastq files derived from WGBS. These files were obtained from online databases: the Sequence Read Archive, ENCODE, and the Genome Sequence Archive. The data were collected from as many different cell types as possible in both humans and mice. Datasets with a maximum read length of less than 50 bases were excluded. To avoid potential bias from different analysis methods, processed sequencing data deposited in the Gene Expression Omnibus were not used. In addition, to avoid potential bias, data generated by reduced-representation bisulfite sequencing and methods based on affinity enrichment and microarray analysis were excluded.

### Generation of methylome maps

Human (hg19) and mouse (mm10) reference genome sequences, along with related metadata such as CpG islands, were obtained from the UCSC Genome Browser. Raw fastq files were processed by trimming low-quality base sequences (<Q30) from the 3′ end of the reads. The remaining high-quality reads, over 50 bases, were then aligned to the reference genomes using Bismark v0.10.0 (Krueger & Andrews, 2011). The following alignment parameters were used: seed length of 28, the maximum number of mismatches in the seed of 1, “--pbat” for post-bisulfite adapter tagging sequence, and “--non-directional” for single-cell bisulfite sequence. Methylation analysis was limited to uniquely aligned reads. Only ACG/TCG sites in NOMe-seq and scCOOL-seq data were analyzed for CpG methylation. For the single-cell bisulfite sequencing dataset, CpG methylation levels were calculated by aggregating single-cell methylation data. CpG methylation levels were determined by combining counts from both strands, and only autosomal CpGs were examined. Global CpG methylation levels in a single sample were calculated by summing the number of methylated and unmethylated CpGs in autosomes for each CpG with a depth of five or greater. Non-CpG methylation was not considered.

### DNA methylation analysis

Because direct WGBS data from human zygotes immediately after fertilization are not currently available, the methylome map of human zygotes at diploid genome formation was estimated using WGBS data from human sperm and oocytes (Okae et al, 2014). After equalizing the sequencing depth in sperm and oocytes at each CpG site, the methylation level of each CpG site was calculated using the number of methylated and unmethylated CpGs in sperm and oocytes. This analysis was restricted to CpG sites where at least four reads were mapped in both sperm and oocytes.

A bin size of 500 kb was chosen to classify megabase-scale methylome patterns. This approach was designed to ensure that methylation levels could be confidently determined even in datasets with low sequence depth, while capturing megabase-scale DNA methylation changes. The procedure for classifying megabase-scale methylome profiles into three classes is described in Fig S1A. Genomic regions with distinct methylation levels were identified using a sliding window method, dividing the genome into 10-kb bins and calculating the total CpG methylation level in each bin. Bins with CpG methylation levels below or above the global CpG methylation level of the sample were classified as small PMDs or small HMDs, respectively. Adjacent small PMDs were combined into a single PMD, whereas adjacent small HMDs were combined into a single HMD. Only PMDs and HMDs larger than 100 kb were analyzed. The oocyte lineage was excluded from PMD analysis because of the nearly complete demethylation of hypomethylated regions.

We examined the behavior of 500-kb genomic bins through the stages of global DNA demethylation and methylation in different lineages, as shown in Fig 3E. These bins were systematically classified into three distinct groups based on their methylation rank (as shown in Fig S1A). At the stage of global hypomethylation (inner cell mass, trophectoderm, and PGCs), the bins were divided into demethylation-resistant (top 1,600 bins), demethylation-intermediate (middle 1,600 bins), and demethylation-susceptible (bottom 1,600 bins) categories. Conversely, at the stage of globally increased methylation (epiblasts, visceral endoderm, trophoblasts, and prospermatogonia), the bins were similarly grouped into methylation-sensitive (top 1,600 bins), methylation-intermediate (middle 1,600 bins), and methylation-resistant (bottom 1,600 bins) classifications. By combining each of these three categories from both the stages of global hypomethylation and global increased methylation, we defined nine distinct groups to analyze the overall dynamics of methylation changes.

Annotations for mouse constitutive LADs were obtained from the GEO database (accession number GSE17051) and subsequently converted to mm10 coordinates. All box and violin plots in the figures were generated using R v3.6.0. As noted in Discussion, there are slight variations in methylation levels between samples of the same tissue and cell type because of batch effects, but these variations were not adjusted for in our analysis.

## Supplementary Material

Reviewer comments
